# Interdiscliplinary Perspectives on Aging at Home Alone With Neurocognitive Impairment

**DOI:** 10.1093/geroni/igaa057.2022

**Published:** 2020-12-16

**Authors:** Laura Girling, Patrick Doyle

## Abstract

Nearly 48 million individuals worldwide have a neurocognitive disorder with projections estimating that as many as 75 million may be afflicted by 2050.Although approximations vary, a substantial portion of those affected live in the community alone, accounting for up to one-third of cases. The true proportion of persons with neurocognitive disorders living alone in the community may be underestimated as dementias are often underdiagnosed and underreported. As the baby boom generation ages and trends towards nuclear families, geographic dispersion of families, and fewer children continue, the number of live-alone persons with neurocognitive impairment is anticipated to rise; creating increased potential for difficult, ambiguous circumstances involving the rights and needs of this population. Despite these trends, available information about this population remains limited. This symposium represents papers from social gerontology, bioethics, and policy; offering unique, but complimentary perspectives on live-alone persons with neurocognitive impairment. The four papers explore 1) how non-traditional & absent support networks impact one’s ability to live alone with dementia [NIA funded], 2) social isolation and vulnerabilities of living alone with dementia [NIA-funded], 3) how bioethics can inform gerontological dementia research [NIA bioethics supplement], and 4) exploration of how law enforcement and adult protective services policies influence the precarity of living alone with dementia. Together, these papers illuminate the importance of actively including live-alone persons with dementia into research and assessing this overlooked vulnerable population from multiple research perspectives (social science, policy, bioethics).

